# Members of the Genus *Methylobacter* Are Inferred To Account for the Majority of Aerobic Methane Oxidation in Oxic Soils from a Freshwater Wetland

**DOI:** 10.1128/mBio.00815-18

**Published:** 2018-11-06

**Authors:** Garrett J. Smith, Jordan C. Angle, Lindsey M. Solden, Mikayla A. Borton, Timothy H. Morin, Rebecca A. Daly, Michael D. Johnston, Kay C. Stefanik, Richard Wolfe, Bohrer Gil, Kelly C. Wrighton

**Affiliations:** a Department of Microbiology, The Ohio State University, Columbus, Ohio, USA; b Department of Soil and Crop Sciences, Colorado State University, Fort Collins, Colorado, USA; c Environmental Science Graduate Program, The Ohio State University, Columbus, Ohio, USA; d Department of Environmental Resources Engineering, State University of New York College of Environmental Science and Forestry, Syracuse, New York, USA; e National Institute of Environmental Health Sciences, Durham, North Carolina, USA; f Department of Civil and Environmental Engineering and Geodetic Sciences, The Ohio State University, Columbus, Ohio, USA; Pacific Northwest National Laboratory; Pacific Northwest National Laboratory

**Keywords:** denitrification, metagenomics, metatranscriptomics, methane, methanotrophs, soil microbiology

## Abstract

Microbial carbon degradation and methanogenesis in wetland soils generate a large proportion of atmospheric methane, a highly potent greenhouse gas. Despite their potential to mitigate greenhouse gas emissions, knowledge about methane-consuming methanotrophs is often limited to lower-resolution single-gene surveys that fail to capture the taxonomic and metabolic diversity of these microorganisms in soils. Here our objective was to use genome-enabled approaches to investigate methanotroph membership, distribution, and *in situ* activity across spatial and seasonal gradients in a freshwater wetland near Lake Erie. 16S rRNA gene analyses demonstrated that members of the methanotrophic *Methylococcales* were dominant, with the dominance largely driven by the relative abundance of four taxa, and enriched in oxic surface soils. Three methanotroph genomes from assembled soil metagenomes were assigned to the genus *Methylobacter* and represented the most abundant methanotrophs across the wetland. Paired metatranscriptomes confirmed that these Old Woman Creek (OWC) *Methylobacter* members accounted for nearly all the aerobic methanotrophic activity across two seasons. In addition to having the capacity to couple methane oxidation to aerobic respiration, these new genomes encoded denitrification potential that may sustain energy generation in soils with lower dissolved oxygen concentrations. We further show that *Methylobacter* members that were closely related to the OWC members were present in many other high-methane-emitting freshwater and soil sites, suggesting that this lineage could participate in methane consumption in analogous ecosystems. This work contributes to the growing body of research suggesting that *Methylobacter* may represent critical mediators of methane fluxes in freshwater saturated sediments and soils worldwide.

## Abstract (precis)

Here we used soil metagenomics and metatranscriptomics to uncover novel members within the genus *Methylobacter*. We denote these closely related genomes as members of the lineage OWC *Methylobacter*. Despite the incredibly high microbial diversity in soils, here we present findings that unexpectedly showed that methane cycling was primarily mediated by a single genus for both methane production (“*Candidatus* Methanothrix paradoxum”) and methane consumption (OWC *Methylobacter*). Metatranscriptomic analyses revealed that decreased methanotrophic activity rather than increased methanogenic activity possibly contributed to the greater methane emissions that we had previously observed in summer months, findings important for biogeochemical methane models. Although members of this *Methylococcales* order have been cultivated for decades, multi-omic approaches continue to illuminate the methanotroph phylogenetic and metabolic diversity harbored in terrestrial and marine ecosystems.

## INTRODUCTION

Wetlands contribute nearly one-third of the naturally derived methane emissions globally, releasing 150 to 250 terragrams of this greenhouse gas per year (1–4). Historically, it was thought that methane was exclusively produced in anoxic horizons of wetland soils by strictly anaerobic methanogenic archaea and was subsequently consumed in oxic zones by aerobic methanotrophic bacteria, with any excess unconsumed methane potentially emitted to the atmosphere (5). These assumptions about microbial methane cycling are incorporated into biogeochemical models that estimate global terrestrial methane budgets (1, 6). However, recent reports of aerobic methanotrophy occurring in hypoxic to anoxic conditions (7–14) and of methanogenesis in oxic soils (15–17) are challenging these historical assumptions. Controlling and accurately forecasting greenhouse gas emissions require more in-depth knowledge of the factors that control natural methane production, consumption, and emission across ecosystems.

To begin to profile biological methane cycling in freshwater wetland soils, we selected the Old Woman Creek (OWC) National Estuarine Research Reserve as our model field site. This 571-acre freshwater wetland borders Lake Erie, near Huron, OH, USA, and has been shown to consistently emit methane (16, 18). During a 5-month period (June through October) in 2015, this wetland emitted approximately 129 million grams of methane and was a net carbon source for the atmosphere during the summer months (18). Previously, it was demonstrated that 40% to 90% of the methane from this wetland was produced in surface soils with oxygenated porewaters by a single methanogen species, “*Ca.* Methanothrix paradoxum” (16). While a taxonomic survey suggested that gammaproteobacterial methanotrophs, i.e., *Methylococcales*, were dominant members throughout the wetland (19), the identity and activity of these methanotrophic microorganisms were not defined along relevant temporal and spatial wetland gradients.

Here we aimed to determine the effects of soil depth, land cover, and season on methanotrophic microorganism distribution and activity in the freshwater wetland. These findings have uncovered genomic information for dominant and highly active methanotrophs within the genus *Methylobacter*, a genus that is present and active in numerous freshwater and marine sediments and in soils (14, 20–23). Given the distribution of this lineage across this wetland, including deeper soils with low dissolved oxygen (DO) concentrations, we analyzed these genomes for potential and active metabolic pathways that could support methane oxidation under hypoxic conditions. Our findings contribute to a growing body of evidence that indicates that the members of the OWC *Methylobacter* lineage are cosmopolitan and active across many freshwater and terrestrial ecosystems.

## RESULTS AND DISCUSSION

### Soil sampling and methane consumption potential.

**FIG 1:**
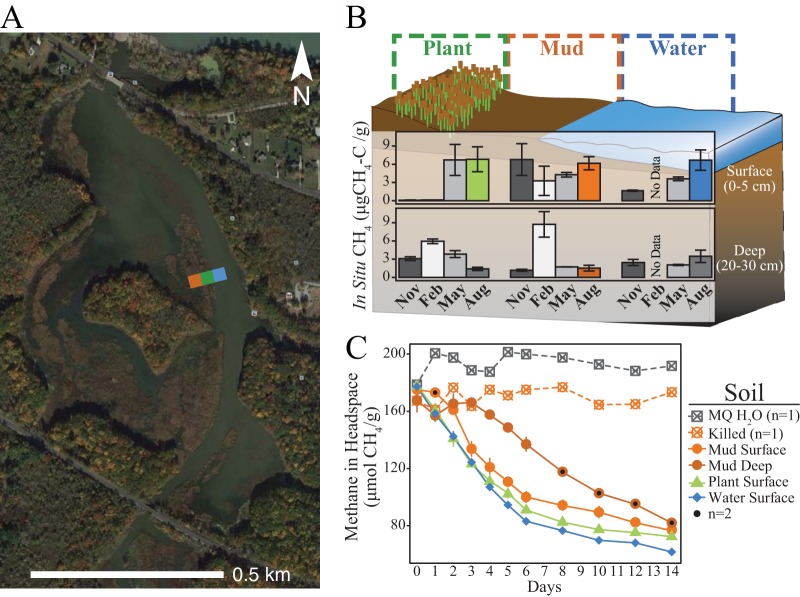
Overview of OWC field site and methane dynamics. (A) Old Woman Creek (OWC) National Estuarine Research Reserve is a 571-acre, NOAA-operated temperate freshwater wetland near Lake Erie in Ohio. Soils were sampled from an ecological transect composed of the following land cover types: *Typha* vegetated (Plant, green), periodically flooded mud flat (Mud, orange), and continually saturated water channel (Water, blue). We selected two soil depths as representative oxic and anoxic soil zones (0 to 5 cm, Surface; 23 to 35 cm, Deep). (B) Soil *in situ* methane concentration variation by depth and land cover type over the four sampled seasons (November 2014 through August 2015). Different months are represented as different shades of gray or are colored by land cover and depth to match the curves shown in panel C. (C) Aerobic methane consumption potential curves of surface and deep soil incubations. Points and curves are colored by the land cover type and depth in the soil column, matching the samples highlighted in panel B.

To understand the impact of seasonality on methanotroph distribution and activity, we sampled soils at four seasonal time points in 2014 to 2015, with sampling occurring in November 2014 representing autumn, February 2015 representing winter, May 2015 representing spring, and August 2015 representing summer. To resolve the impacts of land cover on methanotroph distribution and activity, soils were selected from three land covers (“Plant,” dominated by *Typha* vegetation; “Mud,” periodically exposed mud flats; “Water,” permanently submerged open-water channel sediments) in a transect with locations that were equidistant from Lake Erie (Fig. 1A). At each seasonal time point, from each of the 2-m^2^ land cover plots, three cores were collected for paired 16S rRNA gene analyses. For these analyses, we focused on surface (0 to 5 cm depth) and deep (23 to 35 cm depth) soils (*n* = 66 samples), as these depths were previously demonstrated to have the most distinct bacterial and archaeal communities (19).

During the fall and summer samplings, we conducted chamber measurements, which showed that all of the studied land covers were net methane emitting (16). As a prior study demonstrated (18), eddy-covariance tower measurements showed that the greatest overall methane flux occurred during the summer months of June to September, with the greatest flux peak occurring in August. Compared to the methane emission data, the *in situ* soil methane and dissolved oxygen (DO) concentrations did not differ by season or land cover. However, the levels of both methane and DO decreased with depth across all of the land cover sites (16). The surface soils examined in August had three times more *in situ* methane (6.56 ± 0.83 versus 2.12 ± 0.47 μg CH_4_-C/g) and six times more DO (79.7 ± 11 versus 12.7 ± 7 μM) than the corresponding deep soils (Fig. 1B; see also Data Set S1 in the supplemental material).

10.1128/mBio.00815-18.9 DATA SET S1 Soil geochemistry, 16S rRNA gene amplicon OTU table, genome assembly statistics, metabolic inventory, marker gene comparisons, and biogeography metadata. Download Data Set S1, XLSX file, 2.0 MB. Copyright © 2018 Smith et al. 2018 Smith et al. This content is distributed under the terms of the Creative Commons Attribution 4.0 International license.

To assess the capacity for aerobic methanotrophy in our soils emitting the highest concentrations of methane, August soils were amended with methane and oxygen to measure aerobic methane consumption rates. Methane consumption in surface soils began 3 days sooner than in deep soils and continued at significantly greater rates (Fig. 1C; see also Data Set S1). Methane consumption rates in surface soils were not strongly impacted by land covers (ecological sites) but were likely strongly impacted by *in situ* methane and DO concentrations that varied with soil depth (Fig. 1B) (16). These findings hint that methanotroph activity is likely constrained along centimeters of soil depth rather than in the distinct land covers across meters of lateral distance.

### Members of the *Methylococcales* are the dominant methanotrophs in wetland soils.

**FIG 2:**
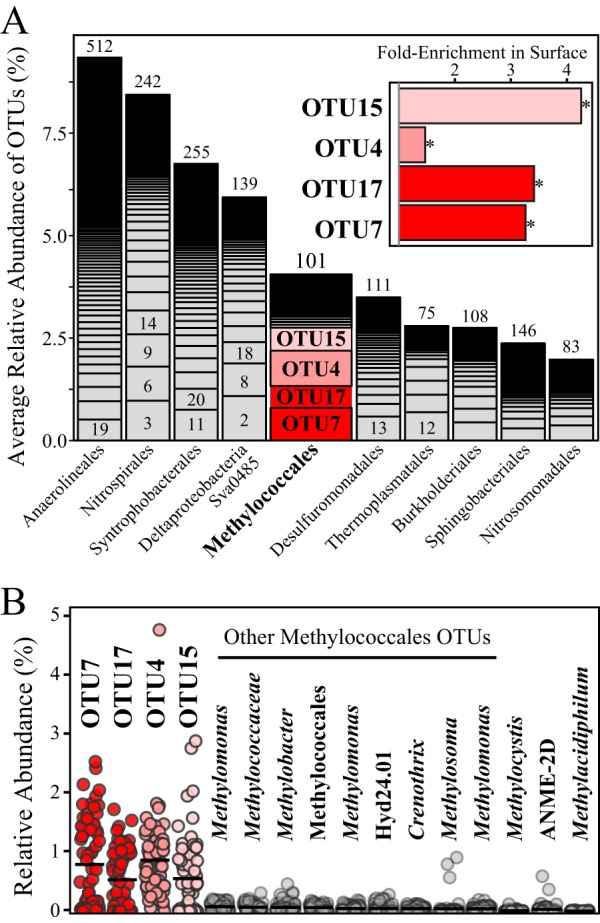
Relative abundances of dominant methanotrophic taxa. (A) Stacked bar chart of the 10 most abundant microbial orders in all soil samples (*n* = 66). The total number of OTUs in each order is noted above the stacked bar chart, and the relative ranks of the 20 most abundant OTUs are indicated. Four dominant *Methylococcales* OTUs are highlighted with shades of red. The inset shows fold enrichment of the dominant OTUs in surface soils over deep soils, with significant differences (analysis of variance with Tukey’s range test; adjusted *P* value [*P*-adj], <0.05) indicated by asterisks. (B) The abundances of the four dominant OTUs compared to those of other detected methane-oxidizing taxa. Shown are the 10 next most abundant *Methylococcales* taxa and the most abundant OTUs of other methanotroph taxa. Dots represent individual samples, and the black bar represents the average. The four dominant OTUs were significantly more abundant (analysis of variance with Tukey’s range test; *P*-adj, <0.05) than all of the other putative methanotrophic OTUs.

The members of the *Methylococcales*, methanotrophs within the *Gammaproteobacteria*, represented the fifth most abundant taxonomic order in all soils collected over four seasons, across three land covers, and at two depths (Fig. 2A). The dominance of this order was largely driven by the relative abundances of four operational taxonomic units (OTUs), which were each among the top 20 most abundant taxa of 5,662 total sampled OTUs (Fig. 2A). Here we denote these dominant methanotroph OTUs by their relative ranks in the microbial community as follows: OTU4 (GQ390219), OTU7 (ABSN01001726), OTU15 (AB5049656), and OTU17 (ABSP01000657). On the basis of the similarity of the 16S rRNA genes (V4 region), these four OTUs were most closely related to an unknown *Crenothrix* species (OTU15), Methylobacter tundripaludum (OTU4), and unassigned *Methylobacter* species (OTU7 and OTU17). On the basis of these partial sequences, OTU7 and OTU17 shared only ∼97% identity with the closest isolated *Methylobacter* representatives M. tundripaludum and Methylobacter psychrophilus. This value is below the recently proposed species cutoff level (98%) for comparison of the V4 regions within members of the *Methylococcaceae* (24); however, we note that caution must be used in interpreting phylogenetic relationships with a single and, especially, partial marker genes.

On average, these four OTUs were each significantly more abundant than all of the OTUs of other known methane-oxidizing taxa (Fig. 2B). Corroborating the methane consumption potential patterns (Fig. 1C), these four OTUs were up to 4-fold more abundant in surface soils than in deeper soils (Fig. 2A, inset) but were not significantly different between land covers or seasons (see Fig. S1A in the supplemental material). Furthermore, the relative abundances of three of these OTUs (OTU7, OTU15, and OTU17) were positively correlated to DO concentrations in the soils (*P* < 0.02) (Fig. S1B). Our findings, along with prior publications from studies of this wetland using data sampled more than a year earlier than here (19), imply that members of the *Methylococcales* are the dominant methanotrophs in surface soils and likely represent critical components of microbial methane cycling in this wetland.

10.1128/mBio.00815-18.2 FIG S1 Dominant *Methylococcales* OTU abundance patterns along spatial, temporal, and geochemical gradients. (A) Fold differences of the OTUs between seasons (left) and sites (right), with significant differences (analysis of variance with Tukey’s range test; *P*-adj, <0.05) indicated by asterisks. (B) Significant correlations (*P < *0.05) between dominant methanotroph OTUs and methane consumption rates (top) and dissolved oxygen concentrations (bottom). The linear relationships are shown only for significant correlations. Download FIG S1, EPS file, 1.0 MB. Copyright © 2018 Smith et al. 2018 Smith et al. This content is distributed under the terms of the Creative Commons Attribution 4.0 International license.

### Discovery and phylogenetic placement of new *Methylococcales* genomes.

To better ascertain the metabolic potential of these dominant *Methylococcales* species in the surface soils, metagenomic sequencing was performed on one representative surface (0 to 5 cm depth) soil from each land cover category (plant, mud, and water) at two time points representing plant senescence in late fall (November 2014) and peak primary productivity (August 2015) (*n* = 6). While we observed no significant differences in methanotroph 16S rRNA gene relative abundances across these gradients, we hypothesized that metagenomics may capture species- or strain-level variations occurring along spatial or seasonal gradients that were not made apparent by 16S rRNA gene sequencing. Additionally, by sequencing metagenomes across various seasons and sites, we expected to increase the likelihood of sampling near-complete genomes from these complex soils, a feature necessary to support our metatranscriptomic analyses.

Metagenomic sequencing yielded 304 Gbp of Illumina HiSeq data. *De novo* assembly of these metagenomes resulted in approximately 3.8 Gbp of genomic information contained in scaffolds greater than 5 kb in size. Using a combination of automated binning and manual binning (see Text S1 in the supplemental material), we recovered four genomic bins likely belonging to methanotrophic bacteria, as determined by the presence of key methanotrophy functional genes and genes with taxonomic affiliation to members of the *Methylococcales*. In accordance with our 16S rRNA gene data (Fig. 2), we did not recover bins for other bacterial or archaeal methanotrophs.

10.1128/mBio.00815-18.1 TEXT S1 Refinement and phylogenetic placement of novel *Methylococcales* genomes and adaptations for low oxygen concentrations. Download Text S1, DOCX file, 0.1 MB. Copyright © 2018 Smith et al. 2018 Smith et al. This content is distributed under the terms of the Creative Commons Attribution 4.0 International license.

The reconstructed methanotroph genomes were estimated to be up to 97% complete (65%, 74%, 81%, and 97%), all with overages of less than 4% (Data Set S1). All of these genomes were from the November metagenomes and would be classified as medium quality using the recently proposed Genomic Standards Consortium benchmarks (25). The August metagenomic sequencing did not yield methanotroph genomes that were greater than 50% complete but did yield other complete genomes, demonstrating that differences in community structure impacted genome recovery.

We recovered three closely related genomes from the different land covers (including genomes NSM2-1 [mud], NSO1-1 [water], and NSP1-1 [plant]), which we conclude are likely members of the same species (discussed below). From one of these genome bins (NSP1-1 scaffold_2426), we recovered a single 404-bp 16S rRNA gene fragment. This gene fragment was 100% identical to all three near-full-length EMIRGE (∼900-bp) (26) sequences generated from unassembled reads from the same November metagenomes where these genomes were recovered (Data Set S1).

Comparison of these near-full-length sequences and the 16S rRNA gene sequences from other *Methylococcales* genomes showed that our recovered metagenome 16S rRNA sequences were closely related strains (>99.8% identity; see Data Set S1) within the genus *Methylobacter*. We, and others (11, 14, 27–30) have noted that the genus *Methylobacter* is not monophyletic and instead contains two (possibly genus-resolved) clades (Fig. S2). Clade 1 contained *Methylobacter* species M. whittenburyi, M. marinus, M. luteus, and M. BBA5.1, while clade 2 contained Methylobacter tundripaludum and M. psychrophilus species. A phylogenetic analysis of our three near-full-length representative OWC *Methylobacter* sequences reconstructed using EMIRGE grouped these genomes with the clade 2 *Methylobacter* genus but were divergent from M. tundripaludum or M. psychrophilus sequences (Fig. S2).

10.1128/mBio.00815-18.3 FIG S2 Phylogenetic placement of EMIRGE 16S rRNA sequences. The maximum likelihood phylogeny represents nearly full-length (trimmed to ∼900-bp) *Methylococcales* 16S rRNA genes and sequences reconstructed using EMIRGE (red), rooted to *Nitrosococcus* species. Colored tips indicate polyphyletic *Methylobacter* (shades of blue) and *Methylomicrobium* (shades of green) clades evident from our multiple phylogenetic analyses and previous publications (see Text S1). Download FIG S2, EPS file, 1.3 MB. Copyright © 2018 Smith et al. 2018 Smith et al. This content is distributed under the terms of the Creative Commons Attribution 4.0 International license.

**FIG 3:**
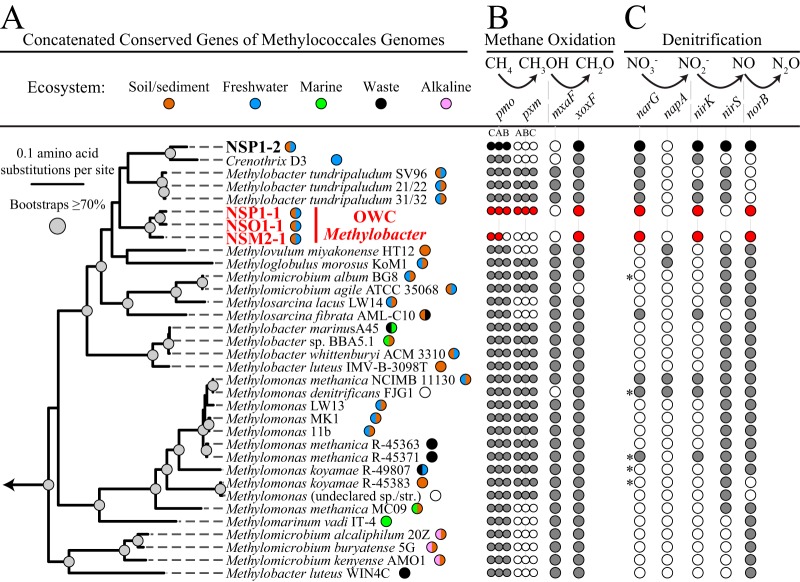
Phylogenetic placement of novel genomes and overview of methane and dissimilatory nitrate reduction metabolisms. (A) Maximum likelihood tree constructed from 21 concatenated universally conserved single-copy and ribosomal protein genes (3,270 amino acids [aa]), rooted to *Nitrosococcus*. Our four genomes represent two novel lineages (Text S1): OWC *Methylobacter* (red) and NSP1-2 (black). Ecosystem types are indicated by colored circles next to the genome name. (B) Inventory of the presence and types of particulate methane monooxygenase (*pmo*), *pmo*-like (*pxm*), and methanol dehydrogenase (*mxaF*, *xoxF*) genes in *Methylococcales* genomes (Data Set S1). (C) Dissimilatory nitrate and nitrite reduction marker genes found in *Methylococcales* genomes. Gene expression or biochemical transformations demonstrating dissimilatory nitrate or nitrite reduction are indicated by the asterisks (Data Set S1). Inventories are not shown for NSO1-1 due to lack of core genes.

Additional phylogenetic analyses using single and concatenated housekeeping genes, as well as single functional genes, confirmed the placement of our genomes within clade 2 and yet also showed that the OWC genomes were divergent from the currently isolated species (M. tundripaludum and M. psychrophilus). For instance, a concatenated phylogenetic tree composed of 14 ribosomal proteins and 7 universally conserved single-copy marker genes that were present in our *Methylococcales* genomes and in 53 other sequenced *Methylococcales* genomes (Fig. 3A) revealed that three of these OWC genomes formed a well-supported lineage that was most closely related to but likely divergent from M. tundripaludum. The results from our *pmoA* gene (Fig. S3), methanol dehydrogenase (Fig. S4), and whole-genome-wide nucleotide and amino acid comparisons (Text S1) also support the characterization of our genomes as members of *Methylobacter* clade 2 and, potentially, as a separate species-level lineage within this genus. We conservatively refer to these genomes at the genus level, denoting that the members of the OWC *Methylobacter* represent a lineage of *Methylobacter* clade 2.

10.1128/mBio.00815-18.4 FIG S3 Phylogenies of *pmoA* nucleotides (left) and amino acids (right). The maximum likelihood tree represents full-length nucleotide (744-bp) and amino acid (247-aa) sequences of *pmoA* genes and gene products in sequenced *Methylococcales* genomes, rooted to *Nitrososcoccus* species’ ammonia monooxygenase (not shown). Bolded red and black tips indicate the OWC *Methylobacter* and NSP1-2 genomes, respectively. Other colored tips indicate the polyphyletic *Methylobacter* (shades of blue) and *Methylomicrobium* (shades of green) clades evident from our multiple phylogenetic analyses and previous publications (Text S1), matching Fig. S2. Download FIG S3, EPS file, 1.3 MB. Copyright © 2018 Smith et al. 2018 Smith et al. This content is distributed under the terms of the Creative Commons Attribution 4.0 International license.

10.1128/mBio.00815-18.5 FIG S4 Types of methanol dehydrogenase encoded by OWC *Methylobacter* and NSP1-2. The maximum likelihood tree represents *xoxF*-type and *mxaF*-type methanol dehydrogenase amino acid sequences rooted to the three unclassified sequences. Clades lacking *Methylococcales* representatives (e.g., all of the *xoxF1*, *xoxF2*, *xoxF4* clades and portions of the *mxaF*, *xoxF3*, and *xoxF5* clades) are collapsed for clarity, and the numbers of representatives collapsed are indicated in parentheses. In the *mxaF* clade, the *xoxF3* clade, and an unassigned *xoxF* clade, the nearest branching neighbors to methanotrophs are colored gray. Members of the *Methylococcales* are bolded. OWC *Methylobacter* and NSP1-2 genes are highlighted in red and black text, respectively. Download FIG S4, EPS file, 1.4 MB. Copyright © 2018 Smith et al. 2018 Smith et al. This content is distributed under the terms of the Creative Commons Attribution 4.0 International license.

A fourth recovered *Methylococcales* genome (NSP1-2) was phylogenetically distinct from the three OWC *Methylobacter* genomes (NSM2-1, NSO1-1, and NSP1-1) (Fig. 3). This more divergent genome, which lacked a 16S rRNA gene recovered from the genome bin, lacked confident taxonomic assignment using our concatenated marker gene phylogenies (Fig. 3A) and *pmoA* phylogenies (Fig. S3; see also Text S1). But this genome appeared most closely related to *Crenothrix* sp. D3 by the use of multiple phylogenetic markers, including concatenated and single-copy marker genes (Fig. 3A; see also Fig. S3 and Text S1). Given this lack of taxonomic congruency and the inability to link to our 16S rRNA gene amplicon data, we focus our primary analyses in the manuscript on the OWC *Methylobacter* clade 2 lineage genomes (NSM2-1, NSO1-1, and NSP1-1).

The discovery of phylogenetic novelty is consistent with recent sampling of the uncultivated diversity within the *Methylococcales* over the past few years. Much of this new insight can be attributed to the reconstruction of genomes from metagenomes obtained from diverse environments (Fig. 3A). This includes the recovery of genomes representing *Methylothermaceae* sp. B42 genome from deep-sea hydrothermal vents (12), the OPU3 genome from marine oxygen minimum zones (10), *Crenothrix* sp. D3 genome from lacustrine waters (11), and Upland Soil Cluster γ from Antarctic cryosols (31). Although *Methylococcales* species have been cultivated for decades, genomes reconstructed from metagenomes continue to illuminate the methanotroph genome diversity present across terrestrial and marine ecosystems.

### OWC *Methylobacter* and the NSP1-2 genomes encode mechanisms to putatively withstand oxygen limitation.

All four of our *Methylococcales* genomes have the essential genes for methane oxidation, including genes encoding particulate methane monooxygenase (*pmo*) (Fig. 3B) and the methanopterin-linked C1 transfer pathway and formate dehydrogenase and the genes necessary for the carbon assimilation via the ribulose monophosphate pathway (RuMP) cycle (Data Set S1). Despite the prevalence of phylogenetic marker genes in the NSO1-1 genome (indicated by its inferred 81% completion), we noted that many core metabolic genes were not recovered in this genome bin. Because we cannot easily distinguish ineffective binning in this metagenome-reconstructed genome from the absence of genes, we do not include a summary of the metabolic potential for this genome in Fig. 3, but the metabolic data for this genome were inventoried (Data Set S1).

Our four genomes encode canonical methane oxidation, aerobic electron transport chain components, and formaldehyde metabolism conserved in other *Methylococcales* (Data Set S1). We failed to detect a soluble methane monooxygenase gene (*smo*) in any of our four genomes; OWC *Methylobacter* genomes likely have the sequence-divergent *pmo* gene (*pxm*) (Fig. 3B; see also Data Set S1). Our reconstructed genomes contained *xoxF5*-type methanol dehydrogenases, but we failed to detect the traditional *mxaF*-type methanol dehydrogenase gene in our genome bins (Fig. 3B; see also Fig. S4) or in any of the unbinned scaffolds in our metagenomic data. Consistent with our findings, the lack of *mxaF* has been reported in methylotrophic microorganisms found in a variety of habitats (10, 32–38). However, we recognize that caution must be used for inferring metabolic capacity on the basis of the absence of genes in genomes derived from metagenomic reconstruction. We also recovered high-affinity cytochrome *bd* ubiquinol oxidase (*cyd*) and Na(+) translocating NADH-quinone oxidoreductase (*nqr*) genes. The functions of some of these genes in methane oxidation are still uncertain, but they may mediate responses to fluctuating oxygen conditions (*cyd*) (9, 39, 40), alter metal requirements or interactions with other community members (*xoxF*) (32, 33, 41), or provide alternative routes for ATP generation via a sodium motive force (*nqr*) (12, 42).

On the basis of recent expansions of the metabolic capacity of *Methylococcales* genomes (43, 44), we inventoried the denitrification potential in our genomes and across the order (Fig. 3C; see also Data Set S1). Our analyses expanded upon research by Padilla et al. indicating that inventoried nitrate reduction potential in 26 members of this order (10). Here we included 31 additional genomes, with a focus on *Methylobacter* members, and also included a survey of methane monooxygenase and methanol dehydrogenase diversity in this order (Fig. 3B; see also Data Set S1). Few of these features appear strongly phylogenetically conserved at the genus level, but major functional differences among *Methylomicrobium* and *Methylobacter* groups were observed. For example, *Methylomicrobium* species most similar to *Methylosarcina* possessed *pxm* whereas the other *Methylomicrobium* species did not (Fig. 3A), and clade 2 but not clade 1 *Methylobacter* species have the capacity for dissimilatory nitrate reduction (Fig. 3C; see also Fig. S5 and Data Set S1). In addition to clade 2 *Methylobacter* species, our analyses revealed the presence of dissimilatory nitrate reduction pathways in over one-third of the sequenced *Methylococcales* genomes (23/57 analyzed) (Fig. 3C; see also Data Set S1). Furthermore, nearly two-thirds of these genomes contained a form of dissimilatory nitrite reduction and nitric oxide reductase (40/57 with *nirK* or *nirS* and 41/57 with *norB*; see Data Set S1). Both of the metabolically more complete OWC *Methylobacter* genomes (NSM2-1 and NSP1-1) and the divergent genome (NSP1-2) contained key functional genes for dissimilatory reduction of nitrate (*narG*), nitrite (*nirK*), and nitric oxide (*norB*) (Data Set S1).

10.1128/mBio.00815-18.6 FIG S5 Characteristics of OWC *Methylobacter* and NSP1-2 *narG* genes. (A) Alignment of *narG* amino acid sequences with poorly conserved residues (shown in gray) and cofactor or substrate binding sites highlighted. OWC *Methylobacter* and NSP1-2 are emphasized in bold red and black. (B) Predicted structures of November mud scaffold_736_gene_16 (orange; qualitative model energy analysis 6 [QMEAN6] value, 0.697), which shares 100% identity with the fragmented *narG* amino acids on NSM2-1 scaffold_238 and on NSP1-2 scaffold_2268_gene_2 (green, QMEAN6 = 0.699) modeled to E. coli NarG crystal structure (gray, PDB code 1q16.1.A). (C) Maximum likelihood tree of full-length *narG* amino acid sequences containing the sequences examined as described for panel A, other *Methylococcales* (black), additional methanotrophs (black), and known nonmethanotrophic denitrifying taxa (gray) and sequences similar to those of divergent *Methylococcales narG* (gray), rooted to archaeal representative Haloferax volcanii (not shown). OWC *Methylobacter* and NSP1-2 are highlighted in bolded red and black text, respectively. Visualization is simplified by collapsing the monophyletic clades, with the number of representatives shown in parentheses. *Methylococcales* spp. with demonstrated *narG* gene expression or with reduced nitrate to nitrous oxide are denoted with asterisks (Data Set S1). Download FIG S5, PDF file, 0.4 MB. Copyright © 2018 Smith et al. 2018 Smith et al. This content is distributed under the terms of the Creative Commons Attribution 4.0 International license.

While some of the recent discoveries of denitrification pathways encoded by *Methylococcales* have noted that the *narG* genes were most phylogenetically related to other bacterial lineages (10, 12), our OWC *Methylobacter narG* genes formed a monophyletic clade with sequences with other *Methylococcales* genomes (Fig. S5C). Moreover, the OWC *Methylobacter narG* sequences contained the necessary residues for substrate and cofactor binding (Fig. S5A) (45) and were structurally homologous to the NarG used for denitrification by Escherichia coli (Fig. S5B). The net impact of this nitrogen-based metabolism is uncertain, as our analyses showed that all of the genomes in our study, and others within this family, lack the capacity to reduce nitrous oxide (*nosZ*). Thus, this proposed denitrification activity could potentially generate nitrous oxide, emitting a more potent greenhouse gas than carbon dioxide or methane (46). While expression of *Methylococcales* denitrification pathways has been observed under laboratory conditions (7–9) and in hypoxic marine systems (10), field-scale studies determining the extent and climatic tradeoffs of this process in terrestrial systems are currently not known.

Given the detection of OWC *Methylobacter* OTUs (OTU7 and OTU17) in deeper hypoxic or anoxic soils (Fig. 2A, inset) (19), we examined our genomes for other mechanisms that would enable greater tolerance of low oxygen and methane concentrations. Prior publications have reported microaerobic fermentation by Methylomicrobium buryatense, another member of the *Methylococcaceae*. In this fermentative metabolism, transformation of formaldehyde through the RuMP and glycolysis to produce pyruvate ultimately leads to mixed acid fermentation products and ATP (11, 47, 48). Similar metabolic capabilities were detected in OWC *Methylobacter* genomes and NSP1-2 (Text S1; see also Data Set S1). However, we acknowledge that it is challenging to infer facultative fermentative metabolism from genomes corresponding to respiratory capacities. In a second example, bidirectional [NiFe] hydrogenase (*hox*) genes were harbored in these genomes, suggesting that hydrogen may be used as an electron donor, as previously reported for more distantly related methanotrophs (49, 50). Lastly, we found hemerythrin genes in our genomes that could be involved in responding to variations in oxygen concentrations or in shuttling oxygen directly to the particulate methane monooxygenase enzyme complex (Text S1) (51–56). In support of the idea of these roles, it was recently shown that the presence of Methylomicrobium buryatense increased the expression of *hox* and hemerythrin genes in response to oxygen starvation (48). From our work and that of others performed across a range of ecosystems, there is increasing evidence that members of the aerobic *Methylococcales* encode multiple mechanisms to sense and maintain methane consumption during oxygen limitation. We posit that this versatile genetic repertoire involved in responses to changes in oxygen concentrations may contribute to the cosmopolitan distribution of these taxa observed under various redox conditions.

### OWC *Methylobacter* genomes are the most active methanotrophs in the oxic wetland soils.

**FIG 4:**
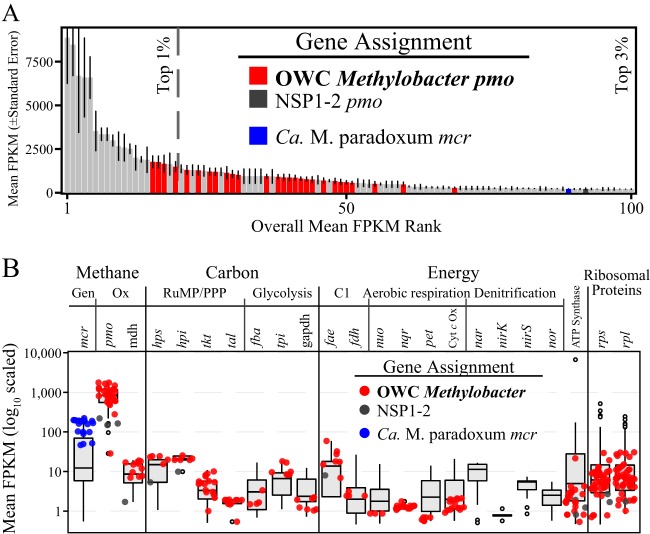
OWC *Methylobacter* and NSP1-2 gene expression in surface soils. (A) Rank abundance curve of the top 100 annotated genes by average normalized gene expression (FPKM [fragments per kilobase of exon per million mapped reads]) in surface soils (*n* = 12). The approximate positions of the top 1% and 3% of the 22,219 genes with detectable transcripts are indicated. (B) Box plots of the mean expression levels of representative genes from core methane (Gen, generation; Ox, oxidation), carbon (RuMP/PPP, ribulose monophosphate pathway or pentose phosphate pathway), and energy generation (C1, C1-transfer) pathways and bulk ribosomal protein gene transcript abundances. The data from the genes assigned to OWC *Methylobacter* and NSP1-2 and the *mcr* gene of “*Ca.* Methanothrix paradoxum” were averaged across all 12 samples and overlaid onto the box plots (colored circles; see Data Set S1). Abbreviations are as follows: *mcr*, methyl coenzyme-M reductase; *pmo*, particulate methane monooxygenase; mdh, methanol dehydrogenase; *hps*, hexulose-phosphate synthase; *hpi*, hexulose phosphate isomerase; *tkt*, transketolase; *tal*, transladolase; *fba*, fructose 1,6-bisphosphate aldolase; *tpi*, triose phosphate isomerase; gapdh, glyceraldehyde phosphate dehydrogenase; *fae*, formaldehyde-activating enzyme; *fdh*, formate dehydrogenase; *nuo*, NADH dehydrogenase; *nqr*, Na(+)-translocating NADH:ubiquinone oxidoreductase; *pet*, ubiquinol cytochrome *bc* reductase; Cyt c Ox, cytochrome *c* oxidase; *nar*, respiratory nitrate reductase; *nirK*, copper-containing nitrite reductase; *nirS*, cytochrome *cd_1_* nitrite reductase; *nor*, nitric oxide reductase; *rps*, small subunit ribosomal protein; *rpl*, large subunit ribosomal protein.

To examine methanotrophic activity among the land covers during different seasons, metatranscriptomic sequencing was performed on triplicate surface soils from the plant and mud land covers in November and August (*n* = 12), yielding 462 Gbp of data (16). OWC *Methylobacter* genomes’ *pmo* genes were among the top 3% most highly transcribed genes in the soils (Fig. 4A) and accounted for nearly 98% of the *pmoA* transcripts (Fig. 4B). The remaining ∼2% of the *pmoA* transcripts were assigned to the divergent NSP1-2 genome (Fig. S6A). Ribosomal protein gene transcript abundances confirmed that OWC *Methylobacter* methanotrophs were some of the most active microorganisms within the surface soil community (Fig. 4B) and that the high transcript abundances were not an artifact of *pmoA* transcript stability. In summary, our data identified members of the OWC *Methylobacter* lineage as the most active methanotrophs in these surface soils.

10.1128/mBio.00815-18.7 FIG S6 Expression of *pmoA* in November and August surface soils (*n* = 12). (A) Stacked bar chart showing the relative contributions of OWC *Methylobacter* and NSP1-2 genotypes to the total *pmoA* levels detected in the metatranscriptomes, averaged across seasons and land covers. (B) Normalized transcript abundances (expressed in fragments per kilobase exon per million reads [FPKM]) of *pmoA* genes recovered in OWC *Methylobacter* and NSP1-2 genomes, averaged across seasons and land covers. (C) Normalized transcript abundances (FPKM) of *mcrA* genes recovered in “*Candidatus* Methanothrix paradoxum” genomes, averaged across seasons and land covers. Insets show the reduction in activity between seasons for the marker genes; the colored text indicates the data shown in the stacked bar charts, and asterisks denote a significant change between seasons (analysis of variance with Tukey’s range test; *P*-adj, <0.05). Download FIG S6, EPS file, 1.1 MB. Copyright © 2018 Smith et al. 2018 Smith et al. This content is distributed under the terms of the Creative Commons Attribution 4.0 International license.

Transcripts for pathways downstream of methane oxidation, e.g., pathways corresponding to methanol dehydrogenase and assimilatory and dissimilatory formaldehyde metabolisms, glycolysis/gluconeogenesis, and aerobic respiration, were also detected for the OWC *Methylobacter* genomes (Fig. 4B). Genes that were notably absent in our metatranscriptomic analyses included genes corresponding to pathways supporting methane oxidation under hypoxic conditions, despite detectable transcripts for a variety of anaerobic metabolic pathways employed by other microorganisms. For example, we did not detect transcripts for OWC *Methylobacter*-catalyzed denitrification (Fig. 4B), the high-affinity terminal oxidase (*cyd*), putative microaerobic fermentation to lactate or ethanol, or hemerythrin by OWC *Methylobacter* in these oxic surface soils (Data Set S2). It is possible that the dissolved oxygen levels in the surface soils (79.7 ± 11.3 μM) precluded the need for oxygen-conserving metabolisms. Ongoing transcript measurements along finely resolved depths will better evaluate the potential activity of these oxygen-conserving mechanisms employed by OWC *Methylobacter* in these soils.

10.1128/mBio.00815-18.10 DATA SET S2 Annotatable genes from assembled metagenomes on contigs of >1 kb detected over 12 surface soil metatranscriptome samples. Download Data Set S2, XLSX file, 2.5 MB. Copyright © 2018 Smith et al. 2018 Smith et al. This content is distributed under the terms of the Creative Commons Attribution 4.0 International license.

A quantitative analysis of the *pmoA* genes recovered in our genomes across wetland gradients revealed a putative seasonal response. While the transcript abundances of OWC *Methylobacter pmoA* genes did not significantly differ between plant and mud land covers in a season, we detected an approximately 4.5-fold decrease in relative transcript abundances from November to August (Fig. S6B). A similar trend was observed for most OWC *Methylobacter* genes (Data Set S2), suggesting that overall methanotrophic metabolism, and not just that of *pmoA* transcripts, was reduced in August. We confirmed that this decrease in inferred activity in August occurred after normalization and thus was not due to seasonal variations in metatranscriptomic sequencing (16). We additionally verified that the decrease in August was not due to a shift in the active methane-oxidizing bacteria by mapping these metatranscriptomes to a database containing 99 *pmoA* genes from sequenced genomes (53 *Methylococcales*, 30 Rhizobiales, 13 *Methylacidiphilum*, and 3 “*Candidatus* Methylomirabilis” genomes, not shown). We entertain the idea that perhaps the OWC *Methylobacter* methanotrophs are cold adapted, similarly to what has been reported for other related *Methylobacter* clade 2 members (14, 21, 29, 57–67). In contrast to the methanotroph activity primarily exhibited by OWC *Methylobacter*, levels of transcripts of normalized methyl coenzyme A reductase (associated with *mcrA*, the functional marker for methanogenesis) from the dominant methanogens did not significantly change between November and August (Fig. S6C) (16). This transcript pattern provides evidence that reduced methanotrophic activity, rather than increased methanogenic activity, may contribute to the increased methane emissions reported in summer months (18). Consequently, this diminished methanotroph activity may also contribute to the ∼2.3-fold-greater *in situ* methane concentrations observed in August surface soils (Fig. 1B).

We previously reported that methane is produced in bulk oxygenated surface soils and that the production is largely mediated by a single methanogen species, “*Ca.* Methanothrix paradoxum” (16). Here we show that OWC *Methylobacter* OTUs (OTU7 and OTU17) and the OTU representing the methanogen “*Ca.* Methanothrix paradoxum” (CU916150) significantly co-occurred in both the mud land cover and plant land covers (*P < *0.02). In the mud land cover, where a disproportionately large quantity of methane is released (18), transcript abundances of OWC *Methylobacter pmoA* and “*Ca.* Methanothrix paradoxum” *mcrA* genes were also highly correlated (*P < *0.02). This suggests that these two dominant methane-cycling microorganisms may form a mutualistic relationship, where the methanogenesis by “*Ca.* Methanothrix paradoxum” that we presume occurs in anoxic microsites (16) subsequently feeds methane oxidation by OWC *Methylobacter* in peripheral oxygenated zones. Methane oxidation leads to further local oxygen scavenging, providing a positive-feedback loop to sustain anaerobic methanogenesis in anoxic microsites within bulk-oxygenated surface soils. Furthermore, dominance by a single methanogen species and a single methanotroph species has been observed in other Northern latitude hydric soils such as thawing permafrost (14, 66–68). Therefore, despite the extremely high richness and strain diversity present in soils, parameterizing microbial methane cycling on the ecosystem scale may be simplified to correspond to several tractable microorganisms.

### OWC *Methylobacter* species are present in other methane-emitting, hydric soil ecosystems.

**FIG 5:**
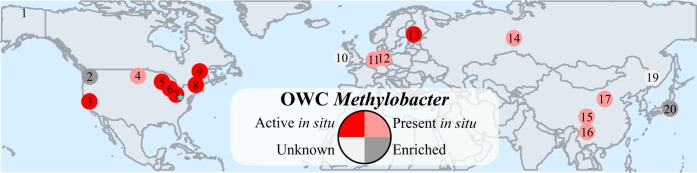
Distribution of OWC *Methylobacter pmoA* genes detected in publicly available sequencing databases. Numbers indicating specific locations are indicated in Data Set S1, and the shading indicates the type of sequencing performed in the study as follows: Active *in situ*, detected in environmental metatranscriptomes; Present *in situ*, detected in environmental metagenomes or clone libraries; Enriched, detected in incubation sequencing studies. NSP1-2 was found at the locations numbered 1, 2, 5, and 7. The cyan star indicates the bubble representing this study. A representation of the sequences assigned to OWC *Methylobacter* and NSP1-2 is visualized in Fig. S7, and the full list of accession numbers and the accompanying metadata are available in Data Set S1.

In an effort to distinguish the global distribution of the OWC *Methylobacter* lineage from that of other closely related *Methylobacter* clade 2 members (M. tundripaludum and M. psychrophilus), we mined publicly available soil and freshwater metagenomic, metatranscriptomic, and clone library databases using *pmoA* genes from OWC *Methylobacter*. We identified 218 of the sequences most closely and significantly affiliated with OWC *Methylobacter* but not with other clade 2 *Methylobacter* members among 71 different sequencing data sets (Fig. S7; see also Data Set S1). Samples containing members closely related to OWC *Methylobacter* were from nine different freshwater and soil locations throughout the United States, Canada, Europe, Russia, China, and Japan (Fig. 5; see also Fig. S7 and Data Set S1). OWC *Methylobacter pmoA* genes were also detected in seven metatranscriptomic studies, suggesting that members of this clade may be active methanotrophs in other ecosystems (Fig. 5; see also Fig. S7 and Data Set S1). These closely related OWC *Methylobacter* genes were found in samples that included Lake Washington sediments where methylotrophic metabolism has been extensively investigated (20, 69) and samples from prairie potholes in North Dakota that showed some of the highest recorded levels of methane fluxes (70) (Data Set S1). Notably, both of the dominant methane-cycling microorganisms present in the OWC soils, “*Ca.* Methanothrix paradoxum” and OWC *Methylobacter*, were present and active in a restored wetland in the San Joaquin Delta in California (16, 71) (Data Set S1), signifying that these two lineages may operate together in other hydric soil systems.

10.1128/mBio.00815-18.8 FIG S7 Simplified depiction of OWC *Methylobacter* and NSP1-2 *pmoA* genes identified in publicly available environmental sequencing studies. The nucleotide reference tree represents *Methylococcales pmoA* rooted to *Nitrosococcus amoA* (not shown) and public sequence data placements. Metadata provided for each biosample or PopSet where sequences similar to those of OWC *Methylobacter* or NSP1-2 were detected are summarized in the grid below. Analyses were not performed on isolated *Methylococcales* strains, and the metadata are not reported for the reference or unassigned sequences. Confidence data indicate an arbitrary strength of the affiliation with our studied genomes as follows: “H” denotes high-confidence placements onto a genome in at least one of the phylogenies and on the genome or its immediate ancestor (only OWC *Methylobacter*) in the other phylogeny; “M” refers to medium confidence by placement on a genome in either nucleic acid or amino acid phylogenies. Sequencing data refer to the type of sequencing data as follows: T, metatranscriptomic; G, metagenomic; C, clone library. “Represented Hits” data describe the total number of sequences represented by the placement in the figure. “Incubation” indicate treatment of samples: N, none; I, incubation study. “Freshwater” data describe the freshwater source of the samples as follows: G, groundwater; L, lake; W, wetland. “Soil” data describe the soil type as follows: A, agricultural; P, permafrost; D, sediment; S, soil. Download FIG S7, EPS file, 1.4 MB. Copyright © 2018 Smith et al. 2018 Smith et al. This content is distributed under the terms of the Creative Commons Attribution 4.0 International license.

### Summary.

Microorganisms inhabiting permafrost, wetlands, and soils in the Northern hemisphere are predicted to be critical for terrestrial-atmospheric methane exchange (6, 14). Here we reconstructed three genomes belonging to the genus *Methylobacter*. From paired metagenomics and metatranscriptomics data, we infer that this OWC *Methylobacter* lineage represents some of the most abundant and active microorganisms across spatial, depth, and seasonal soil gradients. We demonstrated that the level of transcripts indicative of methane consumption activity had decreased 4.5-fold in our summer samples, potentially contributing to the site-wide increase in the levels of methane surface soil concentrations and emission during this time. Genes and transcripts affiliated with OWC *Methylobacter* were detected in other methane-emitting hydric soils and sediments from North America, Europe, Russia, and Asia. Our results indicate that members of clade 2 *Methylobacter* may be important, cosmopolitan methanotrophs present and active across many ecosystems.

## MATERIALS AND METHODS

### Field sample collection.

Old Woman Creek National Estuarine Research Reserve (41°22′N 82°30′W) is located at the southern edge of Lake Erie. The 571-acre freshwater wetland co-operated by the National Oceanic and Atmospheric Administration (NOAA) and the Ohio Department of Natural Resources is one of 28 coastal sentinel research sites. We collected soils and greenhouse gas emissions during November 2014 (fall), February 2015 (winter), May 2015 (spring), and August 2015 (summer). Greenhouse gasses were collected and analyzed as previously described (16, 18). Four or more soil cores were extracted using a modified Mooring system corer from ∼2 m^2^ of soil at three distinct land covers (18, 72–74): emergent vegetated *Typha* (plant), periodically flooded mud flat (mud), and permanently submerged channel sediments (water). In February, six samples from the water channel could not be collected due to frozen, unstable conditions; hence, the total number soil samples analyzed here was 66 and not 72. Cores were stored on ice in the field until hydraulic extrusion and subsampling were performed (∼2 h). DO was measured along the vertical profile in 5-cm increments using an oxygen dipping probe (DP-Pst3) received with a standalone fiber optics Fibox 4 meter (Presens) (16).

Soils were subsectioned into two depths, 0 to 5 cm (surface) and 23 to 35 cm (deep) below the soil surface, and the samples were allocated into sterile WhirlPak bags for biological and geochemical measurements. Soils used for geochemical measurements were stored at 4°C, and soils used for DNA extraction and RNA extraction were stored at −20°C and −80°C, respectively. The methods used to quantify soil and pore water geochemistry were previously described in detail by Angle et al. (16). *In situ* methane concentrations were measured using a Shimadzu GC-2014 chromatograph.

### Methane consumption potential.

Analyses of the aerobic methane consumption potentials of August soils were conducted using a modified version of an experiment previously described by Chan and Parkin (75). Soils from the surface of each land cover and deep soils from the mud land cover collected in August were selected. Soils (5 g) were added to amber vials (35 ml) and were sparged with N_2_ gas immediately. Autoclaved MilliQ (5 ml) was added to improve homogeneity. The headspace of the vials was flushed with 120 ml of air that had been filtered using a 0.2-μm-pore-size filter, and then 2.5 ml (∼10% of the headspace) was removed and replaced with methane. One additional processed surface soil from the mud land cover was autoclaved three times for 20 min each time to serve as a killed control and to account for nonbiological soil methane oxidation. Additionally, one vial containing only 10 ml of sterile MilliQ water was used as a negative control. Methane in the headspace was sampled daily for 1 week and then every other day for the following week. The headspace volume (5 ml) was injected into a Shimadzu GC-2014 chromatograph, and the volume was replaced with a methane-air mixture (approximately 10:90). Consumption rates were calculated from empirically determined linear portions of each curve (see Data Set S1 in the supplemental material).

### Extraction of nucleic acids and preparation of sequencing data for analyses.

16S rRNA gene analyses were performed on surface and deep soils from triplicate cores from each land cover (plant, mud, and water) over four seasons (November, February, May, and August) (*n* = 66). The V4 regions of the 16S rRNA genes were sequenced at Argonne National Laboratory’s Next Generation Sequencing Facility to generate 2-by-251-bp paired-end reads using a single lane of an Illumina MiSeq system (76). Reads were processed using QIIME to generate OTUs and calculate relative abundances (77). To identify the most abundant taxonomic groups (Fig. 2A), the relative abundances of each OTU were averaged over all samples, and the results were then summed according to the unique bacterial and archaeal orders detected. Fold enrichments on each wetland ecological gradient were calculated by comparing the mean relative abundances of the individual OTUs between land covers or seasons.

For metagenomics, we selected surface soils from a single representative core from each of the three land covers (plant, mud, and water) in two seasons (November and August) (*n* = 6). For metatranscriptomics, we performed RNA extractions from each triplicate core and from two land covers (plant and mud) in both seasons (*n* = 12). The nucleic acid extraction protocol was explained previously (16). Briefly, DNA was extracted from each soil sample using MoBio PowerSoil DNA isolation kits, while RNA was extracted using MoBio Powersoil total RNA isolation kits, both performed following the instructions of the manufacturer. DNA was removed from RNA samples using a DNase Max kit (MoBio), and the results were verified by the use of SuperScript III first-strand synthesis (Invitrogen) and PCR.

Genomic DNA was prepared using a TruSeq Rapid Exome Library Prep kit (Kapa Biosystems), and metagenomes were sequenced at The Ohio State University (November) and the Joint Genome Institute (August) using an Illumina HiSeq system. The methods were described previously (16, 77), but briefly, reads for each metagenome were individually assembled *de novo* using IDBA-UD (78), while gene calling and identification were performed by bidirectional querying of multiple databases (79). Scaffolds of >2 kbp in length were binned by tetranucleotide frequencies using emergent self-organizing maps (ESOM) (79, 80) and were further manually curated by GC, coverage, and taxonomic affiliation (see Text S1 in the supplemental material). Completion of each genome was estimated by analysis of the presence of 31 conserved bacterial genes that generally occur in single copy within microbial genomes by the use of Amphora2 (81). Unassembled reads were used to reconstruct near-full-length 16S rRNA gene sequences using EMIRGE (26).

RNA was prepared at JGI using a TruSeq Stranded Total RNA LT Sample Prep kit (Kapa Biosystems), which includes rRNA depletion and cDNA synthesis steps, and was sequenced using an Illumina HiSeq system to generate 2-by-150-bp paired-end reads. Those reads were quality checked and trimmed in the same manner as the metagenomic reads. Reads were mapped to a database containing genes on assembled scaffolds that were >1 kbp from all six metagenomes using Bowtie2 (82), allowing a maximum of 3 mismatches (16). Transcript abundances were corrected for multimapping and normalized by gene length and library size by the use of Cufflinks (83), resulting in units of fragments per kilobase per million mapped reads (FPKM). Separate read mapping to a database of 99 *pmoA* genes, from sequenced genome representatives of *Methylococcales*, *Rhizobiales*, *Methylacidiphilum*, and “*Ca.* Methylomirabilis” retrieved from the Integrated Microbial Genomes and Metagenomes website (IMG/M) or NCBI (see below), was performed in the same manner.

### Phylogenetic analyses of the genomes and marker genes of methanotroph genomes.

Publically available *Methylococcales* genomes were mined in September of 2017 from the Integrated Microbial Genomes and Metagenomes website (IMG/M [https://img.jgi.doe.gov/]) (84). These genomes were supplemented with that of *Crenothrix* sp. D3 (taxonomy identifier [ID] 1880899) (11) obtained via the National Center for Biotechnology Information (NCBI [https://www.ncbi.nlm.nih.gov/]) and with OPU3 extracted from the supplemental material provided by Padilla et al. (10). *Nitrosococcus* species were used as a phylogenetic root because they are members of the *Gammaproteobacteria* and their hallmark ammonia monooxygenase (*amo*) gene shares evolutionary history with *pmo* (40), allowing the same root microorganisms to be used in all phylogenetic analyses, except analyses of methanol dehydrogenase. Genes were identified in these genomes using BLASTp with an E value threshold of 1e−20, and the resulting sequences were manually curated to remove false positives by analysis of operon architectures, sequence alignment, and FastTree topologies (85). Genes on unbinned contigs were assigned to OWC *Methylobacter* or NSP1-2 genomes for transcriptomic analyses by determinations of shared identity levels of >95% over a minimum of 1,000 bp. The affiliations of the genes of interest on these contigs were additionally verified by alignment with the matching genes in the genomes.

For each analysis, genes were aligned using MUSCLE 3.8.31 (86) and were manually curated in Geneious 7.1.9 (87) to remove end gaps and to adjust poorly aligned regions or sequences prior to concatenation performed using Geneious. Maximum likelihood phylogenetic trees were generated using RAxML 8.3.1 (88) with 100 bootstraps.

*Methylococcales* 16S rRNA gene sequences were retrieved from SILVA (https://www.arb-silva.de/) small-subunit (SSU) 128 RefNR (89) and were supplemented with genes in sequenced genomes in IMG/M. This reference database was dereplicated manually by keeping only those sequences present in genomes of isolates or reconstructed from metagenomes and eliminating multicopy rRNA genes (except those of Crenothrix polyspora). The 16S rRNA gene phylogeny was generated using the GAMMAGTR substitution model.

We sought to confirm the identities of the *pmo* and *pxm* genes present in our methanotroph genomes by analysis of branching patterns in addition to conserved operon architecture (40). Operon architectures were visualized on IMG/M using the “Gene Neighborhoods” tool or by scanning the gene orders for OPU3 and *Crenothrix* sp. D3. The phylogenies of *pmoA*, *pmoB*, and *pmoC* were aligned individually using the respective *amo* genes as outgroups. Unbinned *pmo* and *pxm* genes were assigned to OWC *Methylobacter* or NSP1-2 by a combination of overall shared identities and phylogenetic groupings (Data Set S1). Nucleotide phylogeny data were generated using the GAMMAGTR model with Jukes-Cantor correction (28), and the amino acid phylogeny was constructed using the PROTGAMMAWAG substitution (11).

For concatenated phylogenetic analyses using universally conserved single-copy genes (90) and ribosomal protein genes (91), all protein sequences were individually aligned and curated and then concatenated into a single alignment using Geneious. The genes used are described in Data Set S1. All of the genes were present in approximately single copy in all four of our reconstructed genomes, and reference genomes were included only if they were missing a maximum of one gene. The resulting tree (Fig. 3A) was generated using the PROTCATLG model (77, 91). However, we note that the topology of this tree was maintained regardless of the gene concatenation order, the addition or subtraction of genes and genomes, the substitution model, and similarity to the results of single-gene analyses (i.e. ribosomal protein S3; not shown).

In order to determine the type(s) of methanol dehydrogenase encoded by OWC *Methylobacter* and NSP1-2, we compared their methanol dehydrogenase amino acid sequences to those published in Taubert et al. (92). We included additional *Methylococcales* species in order to inventory the methanol dehydrogenase types in this order, as this has not been previously reported (32, 41, 93). The phylogeny (see Fig. S4 in the supplemental material) was generated using the substitution model determined by ProTest (94). Unbinned portions of the metagenomes were mined for *mxaF*-type and *xoxF*-type methanol dehydrogenases (except those that were associated with fewer than 300 amino acids, which were removed) via BLAST and annotation searches and aligned using MUSCLE software, and the types and phylogenetic associations were analyzed using FastTree 2.1.5 (data not shown) (85).

We analyzed the phylogenetic position of *narG* encoded in our genomes by putting these genes in the context of known denitrifying taxa, other *Methylococcales*, other methanotrophs, and genes of distant taxa retrieved from NCBI that were similar to the divergent *narG* gene identified in some *Methylococcales* species. The phylogeny was generated using the substitution model determined by ProTest. We computationally examined the substrate and cofactor binding residues (45) of inferred peptide sequences to provide additional support for the possible activity of these genes. The putative structures of OWC *Methylobacter* and NSP1-2 *narG* were submitted to SWISS-MODEL (https://swissmodel.expasy.org/) (95) for comparison to model NarG encoded by E. coli (PDB code 1q16.1.A).

### Identification of *Methylococcaceae* OWC *pmoA* sequences in public data sets.

Soil (subset of the terrestrial set) and freshwater (subset of the aquatic set) habitat metagenomes and metatranscriptomes publicly available on IMG/M were searched (February 2017) for genes similar to OWC *Methylobacter* and NSP1-2 *pmoA* genes using the BLASTp function with an E value cutoff of 1e−20. We also mined previous publications emphasizing the importance of M. tundripaludum-like *pmoA* sequences in environmental methane cycling and environmental sequences similar to OWC *Methylobacter* or NSP1-2 genes available on NCBI. These included data from Tveit et al. (14), Liebner et al. (29), Martineau et al. (62), and Samad and Bertilsson (96), which are available as Short Read Archives on NCBI under the following accession numbers: SRA SRR524822 and SRR524823, PopSet 159135051, PopSet 300679917, and PopSet 498541747, respectively. Hits that were fewer than 130 amino acids or 400 nucleotides in length (∼50% the total length) were removed from further analyses.

The combination of these filtered databases totaled 2,941 genes and 2,889 peptides from environmental sequence databases. These sequences were aligned to full-length OWC *Methylobacter* sequences (NSM2-1 and NSP1-1), NSP1-2, and reference *Methylococcales* sequences using MUSCLE 3.8.31. A maximum likelihood phylogenetic tree of the reference sequences was generated using RAxML 8.3.1 with 100 bootstraps for both nucleotide and amino acid alignments and GTRGAMMA and GAMMAWAG (11), respectively. Environmental sequences were computationally assigned to nodes using *pplacer* (97), and the specific position of the placement was determined by identifying the node with the greatest log likelihood. Hits that were placed specifically onto NSM2-1, NSP1-1, or NSP1-2 in at least the nucleotide or amino acid analysis were considered to be affiliated with the OWC *Methylobacter* or NSP1-2 and not with neighboring members. To generate Fig. S7, only the hits following these criteria were reanalyzed with *pplacer* using the same reference tree, and the resuts were appended to their branch placements with *guppy* (97). The initial assignments of the hits obtained using *pplacer* are available in Data Set S1.

### Statistical analyses and visualization.

Statistical analyses and data visualizations, including phylogenies, were performed in R 3.3.2, while the methanol dehydrogenase tree was visualized using the interactive Tree Of Life method (iTOL [http://itol.embl.de/]) (98). Significant differences were detected by analysis of variance with *post hoc* correction for multiple comparisons using Tukey’s honest significant difference tests and were defined as an adjusted *P* value of less than 0.05 computed using the “stats” package (*aov* with *TukeyHSD*). Correlations were significant (and are reported here) only in cases in which the *R* value was less than −0.5 or exceeded +0.5, and a *P* value of less than 0.05 as calculated by the use of the “Hmisc” package (*rcorr*). Relationships among relative abundance, gene expression, and geochemical gradient variables were calculated and visualized by fitting to a simple linear model using quantile regression as part of the “stats” package (*lm*). The positions of environmental sequences assigned to our genomes were extracted using the “ggtree” package (*get.placements*).

### Metagenomic and metatranscriptomic pipelines.

The commands used for metagenomic and metatranscriptomic computations can be accessed via respective repositories on our GitHub page (https://github.com/TheWrightonLab/).

### Accession number(s).

Methanotroph genomes generated here are available on NCBI under the following accession numbers (Data Set S1): SAMN05908750 (NSM2-1), SAMN05908751 (NSO1-1), SAMN05908747 (NSP1-1), SAMN05908748 (NSP1-2). Metagenomes and metatranscriptomes can be accessed via NCBI under the following BioSample numbers: SAMN06267298 (November 2014 plant metagenome), SAMN05892948 (November 2014 water metagenome), SAMN05892929 (November 2014 plant metagenome), SAMN06267290 (August 2015 mud metagenome), SAMN06267291 (August 2015 water metagenome), and SAMN06267292 (August 2015 plant metagenome), and SAMN06267298, SAMN06267299, SAMN06267300, SAMN06267301, SAMN06267302, SAMN06267303, SAMN06267304, SAMN06267305, SAMN06267306, SAMN06267307, SAMN06267308, and SAMN06267309 (November 2014 and August 2015 metatranscriptomes). 16S rRNA gene amplicon sequencing data can be retrieved from NCBI under BioProject PRJNA338276.
