# Supplementary material for: Members of the Genus Methylobacter Are Inferred To Account for the Majority of Aerobic Methane Oxidation in Oxic Soils from a Freshwater Wetland
Source: mBio. 2018 Nov 6;9(6):e00815-18. doi: 10.1128/mBio.00815-18 (PMC6222125; doi:10.1128/mBio.00815-18)
Supplement: TEXT S1 [file mbo005184158s1.docx]

**Text S1:** Refinement and phylogenetic placement of the Methylococcales genomes, and adaptations for low oxygen concentrations.

**Binning and refinement of four methanotroph genomes**

Attempts to automate binning using MetaBAT ([1](#_ENREF_1), [2](#_ENREF_2)) generated methanotroph population bins where the algorithm could not differentiate related closely related genomes in the November Plant and Mud metagenomes. Therefore we used Emergent Self-Organizing Maps (ESOM) ([3](#_ENREF_3)) to distinguish these closely related genotypes reconstructed from the November Plant and Mud metagenomes (Data Set S1). This approach, in addition to genome coverage differences within a single ESOM bin, was used to manually resolve closely related genotypes. Removal of the less abundant, but closely related, strains did negatively impact the overall genome completion estimates but significantly decreased estimated ‘contamination’ within the bins. This approach resulted in the resolution of a NSM2-1 genome that was 74% complete with 0% contamination and a NSP1-1 genome that was 65% complete with 0% contamination (Data Set S1). Binning by ESOM of the November Open water metagenome recovered a single methanotroph genome without strain contamination, yielding NSO1-1 that was 81% complete with 3.2% contamination (Data Set S1). The remaining portions of these methanotroph population bins from Nov Plant and Mud bins were not included in further genome-resolved analyses. However, all the assembled (binned and un-binned) metagenome scaffolds were used for transcript mapping and subsequent activity inferences.

**Other markers not discussed in main text pertaining to position of OWC *Methylobacter* genomes (NSM2-1, NSO1-1, and NSP1-1)**

Prior to the advent of whole genome and metagenomic sequencing, the functional gene particulate methane monooxygenase (*pmoA*) served as one of the primary phylogenetic marker for aerobic methanotrophs predominant in our field site ([4](#_ENREF_4)). We compared the full-length *pmoA* genes recovered in NSM2-1, NSP1-1, and NSP1-2 to full-length genes in other sequenced Methylococcales genomes. By pairwise identity, OWC *Methylobacter* *pmoA* genes (from NSM2-1 and NSP1-1) would be assigned to the same species regardless if the nucleotide or amino acids were compared – their nucleotide sequences were 96.0% similar ([5](#_ENREF_5)) and their amino acid sequences were 98.7% similar ([6](#_ENREF_6)) (Data Set S1). In contrast, OWC *Methylobacter* *pmoA* genes only shared 86.0-86.4% nucleotide identity and 91.9-92.7% amino acid identity with their closet relative *M. tundripaludum*, which are below the suggested species cutoffs for this gene (Data Set S1) ([5](#_ENREF_5), [6](#_ENREF_6)). Construction of phylogenetic trees for both nucleotides and amino acids confirmed pairwise identity observations that OWC *Methylobacter* genomes formed a separate lineage closely related to *M. tundripaludum* (Fig. S3). Topology of the OWC *Methylobacter* *xoxF5* within a methanol dehydrogenase phylogenetic analysis (Fig. S4) reinforces the distinction between the OWC *Methylobacter* and the two other species of *Methylobacter* Clade 2 ([7](#_ENREF_7)). However, we note it is unlikely that a single gene has the resolution to accurately resolve species.

We additionally examined the whole-genome aggregated Average Amino acid Identity (AAI) between Methylococcales, which has been proposed as a method to associate metagenome-assembled genomes with accepted genera if they share >65% AAI, or species if they share >90% AAI ([8](#_ENREF_8)). Skennerton *et. al.*, showed that well-sampled Methylococcales genera shared ~70% AAI ([9](#_ENREF_9)). The OWC *Methylobacter* genomes shared 83-97% AAI with each other, and 76% AAI with *M. tundripaludum* in Clade 2, followed by 72% AAI with Clade 1 *Methylobacter*. Similar to the AAI, Average Nucleotide Identity (ANI) between OWC *Methylobacter* ranged from 87-98% ANI, but shared 78-79% ANI with Clade 2 *Methylobacter* and 76-77% ANI with Clade 1 *Methylobacter*. These analyses corroborate that OWC *Methylobacter* are more related to each other then they are to other *Methylobacter*.

**Recovery and phylogenetic placement of a fourth, divergent Methylococcales genome**

In contrast to our other three genomes, the fourth genome that was also reconstructed from the November plant metagenome (NSP1-2) using only ESOM that resulted in a single, clean genome (Data Set S1). This genome was dissimilar compared to the other three by AAI and phylogenetic analyses, sharing no more than 72% AAI with the other three OWC *Methylobacter* genomes. The NSP1-2 genome was most similar to *Crenothrix* sp. D3 by whole genome AAI (83%) and multiple phylogenetic analyses. For instance, this genome was affiliated *Crenothrix* sp. D3 in the *pmoA* nucleotide, concatenated single copy marker gene, and methanol dehydrogenase trees (Fig. S3, Fig. 3A, Fig. S4). Again, this association generally lacked sufficient bootstrap support to confidently assign this new genome to the *Crenothrix* genus. Because NSP1-2 also lacks a 16S rRNA gene sequence, lacks morphological data, and was distant from Crenothrix sp. D3 in *pmoA* amino acid phylogeny, we hesitate to name NSP1-2 as a member of the *Crenothrix* genus. Furthermore, with only a single genome sequenced representative, we also cannot rule out that this affiliation with *Crenothrix* sp. D3 is an artifact of long branch attraction. Therefore, we refer to this single divergent genome as “Unassigned Methylococcales NSP1-2”. Biogeographic analyses were performed using the *pmoA* present in this genome, which showed that closely related methanotrophs were found across the United States (Fig. 5, Fig. S7, Data Set S1).

**Putative oxygen sensing and facultative fermentation potential by the OWC *Methylobacter* and NSP1-2**

Prior reports have suggested that Methylococcales may be able to sense oxygen using a bacteriohemerythrin protein. A model methanotroph expressed a hemerythrin that interacted with the particulate methane monooxygenase enzyme complex, leading to the hypothesis that it may act as a trace oxygen sensor or may be involved in delivering oxygen directly to the enzyme for methane oxidation ([10-14](#_ENREF_10)). OWC *Methylobacter* and NSP1-2 genomes possessed putative oxygen-sensing hemerythrin genes (>55% amino acid identity to MCA0715, Data Set S1), which contained all of the proposed iron and oxygen binding sites ([13](#_ENREF_13)). This hemerythrin gene is well conserved within the Methylococcales (present in 52/57 of sequenced Methylococcales genomes) suggesting that this gene may serve a critical function, however the physiological role and environmental context for this gene expression is currently limited. A recent publication did find that the expression of a bacteriohemerythrin gene was increased during oxygen starvation ([15](#_ENREF_15)), adding evidence to the hypothesis that this protein may be important for responding to oxygen stress.

Oswald *et al.* inferred the pathway to support methanotrophic micro-aerobic fermentation observed during oxygen-limited growth of *Methylomicrobium buryatense* ([15-18](#_ENREF_15)). They proposed that the glycolytic intermediate pyruvate could be used to produce succinate, acetate, lactate, and hydrogen as mixed-acid fermentation products. OWC *Methylobacter* genomes, rather than producing succinate or acetate, may produce lactate or ethanol by using lactate dehydrogenase or ethanol-aldehyde dehydrogenase, respectively (Data Set S1). We do not expect that these organisms could produce succinate or acetate because the genomes lacked fumarate reductase for succinate production, and both acetate kinase and phosphoacetyltransferase for acetate production. Similarly, NSP1-2 encoded the genes for lactate and ethanol production, but also contained acetate kinase (Data Set S1). OWC *Methylobacter* and NSP1-2 may produce hydrogen using a bidirectional [NiFe] hydrogenase (*hoxFUYH*), however we acknowledge that the function(s) of this hydrogenase in the Methylococcales is unknown. Recently, Gilman *et al.* showed that *hoxFUYH* expression by *M. buryatense* increased during oxygen starvation but no hydrogen was evolved, therefore it was concluded that this hydrogenase may instead be consuming hydrogen ([15](#_ENREF_15)). Distantly related methanotrophic Verrucomicrobia are able to use hydrogen as an electron donor, but the specific hydrogenase(s) involved in this metabolism have yet to be elucidated ([19](#_ENREF_19), [20](#_ENREF_20)). In addition to *hoxFUYH*, we also note that OWC *Methylobacter* and NSP1-2 genomes encoded formate hydrogenlyase (*fhl*) complex known to evolve hydrogen in *E. coli* ([21](#_ENREF_21)), but the role of *fhl* in methanotrophs is also unknown. Compared to the hydrogenases in *E. coli*, the *hox* genes encoded by OWC *Methylobacter* and NSP1-2 are most similar to flexible hydrogenase-2 (*hyb*) that will produce hydrogen under fermentation conditions but consume it under respiring conditions ([22-25](#_ENREF_22)), whereas the *fhl* genes are most similar to hydrogenase-3 (*hyc*) that evolves hydrogen ([21](#_ENREF_21)). The presence of these hydrogenase types suggests that both hydrogen consumption and production may be possible for OWC *Methylobacter* and NSP1-2 genomes, with directionality dependent on oxygen availability. The scaffolds that these genes occur on are available in Data Set S1.

**References**

1. Angle JC, Morin TH, Solden LM, Narrowe AB, Smith GJ, Borton MA, Rey-Sanchez C, Daly RA, Mirfenderesgi G, Hoyt DW, Riley WJ, Miller CS, Bohrer G, Wrighton KC. 2017. Methanogenesis in oxygenated soils is a substantial fraction of wetland methane emissions. Nature Communications 8:1567.

2. Kang DD, Froula J, Egan R, Wang Z. 2015. MetaBAT, an efficient tool for accurately reconstructing single genomes from complex microbial communities. PeerJ 3:e1165.

3. Dick GJ, Andersson AF, Baker BJ, Simmons SL, Thomas BC, Yelton AP, Banfield JF. 2009. Community-wide analysis of microbial genome sequence signatures. Genome biology 10:R85.

4. McDonald IR, Murrell JC. 1997. The particulate methane monooxygenase gene pmoA and its use as a functional gene probe for methanotrophs. FEMS microbiology letters 156:205-210.

5. Wen X, Yang S, Liebner S. 2016. Evaluation and update of cutoff values for methanotrophic pmoA gene sequences. Archives of Microbiology 198:629-636.

6. Knief C. 2015. Diversity and Habitat Preferences of Cultivated and Uncultivated Aerobic Methanotrophic Bacteria Evaluated Based on pmoA as Molecular Marker. Frontiers in Microbiology 6.

7. Lau E, Fisher MC, Steudler PA, Cavanaugh CM. 2013. The Methanol Dehydrogenase Gene, mxaF, as a Functional and Phylogenetic Marker for Proteobacterial Methanotrophs in Natural Environments. PLoS ONE 8:e56993.

8. Konstantinidis KT, Rosselló-Móra R, Amann R. 2017. Uncultivated microbes in need of their own taxonomy. The ISME Journal 11:2399.

9. Skennerton CT, Ward LM, Michel A, Metcalfe K, Valiente C, Mullin S, Chan KY, Gradinaru V, Orphan VJ. 2015. Genomic reconstruction of an uncultured hydrothermal vent gammaproteobacterial methanotroph (family Methylothermaceae) indicates multiple adaptations to oxygen limitation. Frontiers in microbiology 6.

10. Bailly X, Vanin S, Chabasse C, Mizuguchi K, Vinogradov SN. 2008. A phylogenomic profile of hemerythrins, the nonheme diiron binding respiratory proteins. BMC Evol Biol 8:244.

11. Chen KH-C, Wu H-H, Ke S-F, Rao Y-T, Tu C-M, Chen Y-P, Kuei K-H, Chen Y-S, Wang VC-C, Kao W-C. 2012. Bacteriohemerythrin bolsters the activity of the particulate methane monooxygenase (pMMO) in Methylococcus capsulatus (Bath). Journal of inorganic biochemistry 111:10-17.

12. Kao W-C, Wang VC-C, Huang Y-C, Yu SS-F, Chang T-C, Chan SI. 2008. Isolation, purification and characterization of hemerythrin from Methylococcus capsulatus (Bath). Journal of inorganic biochemistry 102:1607-1614.

13. Karlsen OA, Ramsevik L, Bruseth LJ, Larsen Ø, Brenner A, Berven FS, Jensen HB, Lillehaug JR. 2005. Characterization of a prokaryotic haemerythrin from the methanotrophic bacterium Methylococcus capsulatus (Bath). The FEBS journal 272:2428-2440.

14. Schaller RA, Ali SK, Klose KE, Kurtz Jr DM. 2012. A bacterial hemerythrin domain regulates the activity of a Vibrio cholerae diguanylate cyclase. Biochemistry 51:8563-8570.

15. Gilman A, Fu Y, Hendershott M, Chu F, Puri AW, Smith AL, Pesesky M, Lieberman R, Beck DA, Lidstrom ME. 2017. Oxygen-limited metabolism in the methanotroph Methylomicrobium buryatense 5GB1C. PeerJ 5:e3945.

16. Gilman A, Laurens LM, Puri AW, Chu F, Pienkos PT, Lidstrom ME. 2015. Bioreactor performance parameters for an industrially-promising methanotroph Methylomicrobium buryatense 5GB1. Microbial cell factories 14:182.

17. Kalyuzhnaya MG, Yang S, Rozova ON, Smalley NE, Clubb J, Lamb A, Gowda GA, Raftery D, Fu Y, Bringel F, Vuilleumier S, Beck DA, Trotsenko YA, Khmelenina VN, Lidstrom ME. 2013. Highly efficient methane biocatalysis revealed in a methanotrophic bacterium. Nat Commun 4:2785.

18. Oswald K, Graf JS, Littmann S, Tienken D, Brand A, Wehrli B, Albertsen M, Daims H, Wagner M, Kuypers MM. 2017. Crenothrix are major methane consumers in stratified lakes. The ISME Journal.

19. Carere CR, Hards K, Houghton KM, Power JF, McDonald B, Collet C, Gapes DJ, Sparling R, Boyd ES, Cook GM. 2017. Mixotrophy drives niche expansion of verrucomicrobial methanotrophs. The ISME journal 11:2599.

20. Mohammadi S, Pol A, van Alen TA, Jetten MS, den Camp HJO. 2017. Methylacidiphilum fumariolicum SolV, a thermoacidophilic ‘Knallgas’ methanotroph with both an oxygen-sensitive and-insensitive hydrogenase. The ISME journal 11:945.

21. McDowall JS, Murphy BJ, Haumann M, Palmer T, Armstrong FA, Sargent F. 2014. Bacterial formate hydrogenlyase complex. Proceedings of the National Academy of Sciences 111:E3948-E3956.

22. Laurinavichene TV, Chanal A, Wu L-F, Tsygankov AA. 2001. Effect of O2, H2 and redox potential on the activity and synthesis of hydrogenase 2 in Escherichia coli. Research in microbiology 152:793-798.

23. Laurinavichene TV, Tsygankov AA. 2001. H2 consumption by Escherichia coli coupled via hydrogenase 1 or hydrogenase 2 to different terminal electron acceptors. FEMS microbiology letters 202:121-124.

24. Laurinavichene TV, Zorin NA, Tsygankov AA. 2002. Effect of redox potential on activity of hydrogenase 1 and hydrogenase 2 in Escherichia coli. Archives of microbiology 178:437-442.

25. Pinske C, Jaroschinsky M, Linek S, Kelly CL, Sargent F, Sawers RG. 2015. Physiology and bioenergetics of [NiFe]-hydrogenase 2-catalyzed H2-consuming and H2-producing reactions in Escherichia coli. Journal of bacteriology 197:296-306.
