# Supplementary material for: Members of the Genus Methylobacter Are Inferred To Account for the Majority of Aerobic Methane Oxidation in Oxic Soils from a Freshwater Wetland
Source: mBio. 2018 Nov 6;9(6):e00815-18. doi: 10.1128/mBio.00815-18 (PMC6222125; doi:10.1128/mBio.00815-18)
Supplement: FIG S5 [file mbo005184158sf5.pdf]

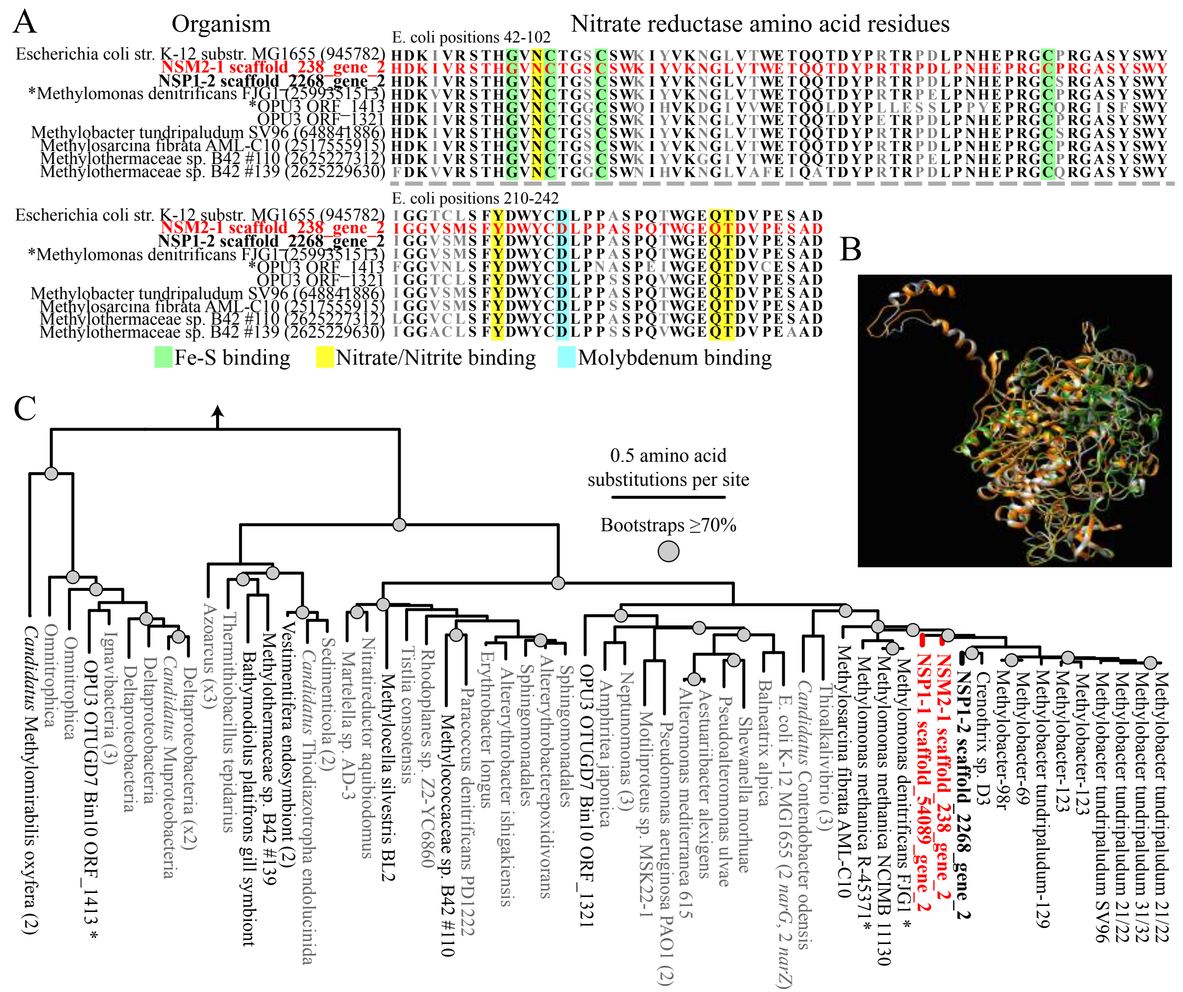

Figure S5. Characteristics of OWC *Methylobacter* and NSP1-2 *narG* genes. (A) Alignment of *narG* amino acid sequences with poorly conserved residues shown in gray, and cofactor or substrate binding sites highlighted. OWC *Methylobacter* an NSP1-2 are emphasized in bold red and black. (B) Predicted structures of Nov. mud scaffold_736_gene_16 (orange, QMEAN6 = 0.697), which shares 100% identity to both fragmented *narG* amino acids on NSM2-1 scaffold_238, and NSP1-2 scaffold_2268_gene_2 (green, QMEAN6 = 0.699) modeled to *E. coli* NarG crystal structure (gray, pdb number: 1q16.1.A). (C) Maximum likelihood tree of full-length *narG* amino acid sequences containing the sequences examined in (A), other Methylococcales (black), additional methanotrophs (black), known non-methanotrophic denitrifying taxa (gray), and sequences similar to divergent Methylococcales *narG* (gray), rooted to archaeal representative *Haloferax volcanii* (not shown). The OWC *Methylobacter* and NSP1-2 are highlighted in bolded red and black text respectively. Visualization is simplified by collapsing monophyletic clades with the number of representatives shown in the parentheses. Methylococcales with demonstrated *narG* gene expression or have reduced nitrate to nitrous oxide are denoted with asterisks (Data Set S1).
